# The Associations Between Seven Different Types of Physical Activity and the Incidence of Fracture at Seven Sites in Healthy Postmenopausal UK Women

**DOI:** 10.1002/jbmr.3896

**Published:** 2019-11-15

**Authors:** Miranda EG Armstrong, Jason Lacombe, Clare J Wotton, Benjamin J Cairns, Jane Green, Sarah Floud, Valerie Beral, Gillian K Reeves, for the Million Women Study Collaborators

**Affiliations:** ^1^ School for Policy Studies, University of Bristol Bristol UK; ^2^ Cancer Epidemiology Unit University of Oxford Oxford UK; ^3^ MRC Population Health Research Unit, Nuffield Department of Population Health University of Oxford Oxford UK

**Keywords:** EPIDEMIOLOGY, EXERCISE, FRACTURE PREVENTION, GENERAL POPULATION STUDIES, OSTEOPOROSIS

## Abstract

There is a paucity of information on associations between specific types of physical activity and fracture risk at different sites in otherwise healthy postmenopausal women. Therefore, we examined risk of fracture at seven different sites associated with seven different types of physical activity in the population‐based prospective UK Million Women Study. A total of 371,279 postmenopausal women (mean age 59.8 years), rating their health as good or excellent and reporting participation in walking, cycling, gardening, doing housework, yoga, dance, and sports club activities, were followed for site‐specific incident fracture through record linkage to national databases on day‐case and overnight hospital admissions. Cox regression yielded adjusted relative risks (RRs) and, because of the large number of statistical tests done, 99% confidence intervals (CIs) for fracture at seven different sites in relation to seven different physical activities. During an average follow‐up of 12 years, numbers with a first site‐specific fracture were as follows: humerus (2341), forearm (1238), wrist (7358), hip (4354), femur (not neck) (617), lower leg (1184), and ankle (3629). For upper limb fractures there was significant heterogeneity across the seven activity types (test for heterogeneity *p* = 0.004), with gardening more than 1 hour/week associated with a lower risk (RR = 0.91; 99% CI, 0.86 to 0.96; *p* < 0.0001), whereas cycling more than 1 hour/week was associated with an increased risk (RR = 1.11; 99% CI, 1.00 to 1.23; *p* = 0.008). For fractures of the lower limb (including hip) there was no significant heterogeneity by type of activity, with significant approximately 5% to 15% reductions in risk associated with most activities, except cycling. For hip fractures, there was no significant heterogeneity by type of activity, but with significant 15% to 20% reductions in risk associated with walking for 1 hour/day and participating in yoga and sporting activities. Physical activity is a modifiable risk factor for fracture, but the effects differ between different types of activities and different fracture sites. © 2019 The Authors. *Journal of Bone and Mineral Research* published by American Society for Bone and Mineral Research.

## Introduction

Fractures are an important cause of morbidity and mortality among postmenopausal women.^1^ Prior research has shown a reduction in the risk of hip fracture with increased physical activity,^2^, ^3^, ^4^, ^5^, ^6^ and clinical trials show physical activity reduces fractures and falls through improvements in balance and muscular strength.^7^, ^8^, ^9^, ^10^ However, there is limited evidence on how physical activity is associated with fracture at sites other than the hip and there is a paucity of information on how a variety of specific physical activities are associated with risk of fracture.^11^, ^12^ Physical activities may improve balance^13^ and muscle strength,^14^ and aid in the preservation of bone mineral density,^15^, ^16^ which could reduce the risk of fracture. Conversely, injury risk may be increased while engaging in physical activity.^17^ Increased automation has resulted in reduced activity requirements in daily life, with a higher proportion of the population spending more time sedentary.^18^ Further, increasing leisure time activity in older adults may be challenging with a lack of interest cited as an important deterrent.^19^ Therefore, determining whether there is an association between fracture and physical activities such as gardening and housework may be as important as assessing activities more commonly measured such as walking, cycling, and other leisure time activities.

In this large, prospective study of healthy postmenopausal UK women, we describe the independent relationships of seven specific physical activities: walking, gardening, housework, cycling, yoga, sports club participation, and dance participation, with the risk of fracture at seven sites: humerus, forearm, wrist, hip, femur (not neck), lower leg, and ankle.

## Subjects and Methods

### Participants and data

The Million Women Study is a large, population‐based prospective study of UK women recruited through 66 National Health Service (NHS) breast cancer screening clinics. In 1996 to 2001, 1.3 million women aged 56 ± 4.8 years (age range, 48 to 90 years) on average, completed a self‐administered questionnaire including details on anthropometry, lifestyle, and other factors. About 3 years later a postal resurvey questionnaire was sent to participants asking, among other factors, about specific physical activities and self‐reported general health, and this questionnaire forms the baseline for this study. Study questionnaires and further details of the data and access policies can be viewed on the website (http://www.millionwomenstudy.org). Full details of the design and study methods have been described elsewhere.^20^ The Oxford and Anglia Multi‐Centre Research Ethics Committee provided ethics approval for this study.

Each woman was linked, using her unique NHS identification number and date of birth, to NHS information on deaths, cancer registrations, and hospital admission databases: Hospital Episode Statistics (HES) for England,^21^ and Scottish Morbidity Records for Scotland.^22^ These databases include information on inpatient stays and day‐case admissions (women admitted and discharged on the same day), but not on outpatient visits. Follow‐up is virtually complete for this study population, with loss to follow‐up of only 1%.^20^

Information on admission and discharge dates, and details of the diagnoses and procedures associated with each hospital admission, were provided, coded to the World Health Organization's International Classification of Diseases, 10th revision (ICD‐10).^23^ Incident cases were defined as the first hospital record (day‐case or overnight admission) of fracture (either primary or secondary diagnosis) of the humerus (S42.2–S42.4), forearm (S52.0–S52.4, S52.7), wrist (S52.5–S52.6, S62.0–S62.1, S62.8), hip (S72.0–S72.1), femur (not neck) (S72.2–S72.4), lower leg (not ankle) (S82.1–S82.2, S82.4), or ankle (S82.3, S82.5–S82.6, S82.8), occurring after analysis baseline. These sites were chosen because they were the most common fracture sites. We have only provided forest plots for individual fracture sites with more than 1000 fractures. Although vertebral fractures account for a high number of fragility fractures, they are difficult to diagnose and are often diagnosed only by chance, because some are asymptomatic or have nonspecific symptoms.^24^ Therefore, because of the trend for high levels of underdiagnosis, we chose not to include vertebral fractures in our site‐specific analyses. Risk of subsequent fracture is increased following a fracture,^25^ and to ensure that this was accounted for we censored our analyses at the first occurrence of any fracture. Our definition of any fracture for censoring purposes included all fracture codes according to ICD‐10, defined as the following ICD‐10 codes: M48.4, M80, M84.3, S02, S12, S22, S32, S42, S52, S62, S72, S82, S92, T02, T08, T10, T12, T14.2, in addition to those fracture codes named above. We considered “upper limb fractures” to include fractures of the humerus, forearm, and wrist, and “lower limb fractures” to include fractures of the hip, femur, lower leg, and ankle.

Menopause has a profound effect on bone mineral density^26^; therefore, we restricted our analyses to postmenopausal women. If women reported being premenopausal, perimenopausal, or of unknown menopausal status at study baseline we assumed they were postmenopausal once they reached the age of 55 years. We made this assumption because 96% of women in this cohort with a known age of natural menopause were postmenopausal by the age of 55 years. Women with Turner's syndrome were excluded, as were those with missing information on physical activity or BMI. We excluded women with a hospital record of fracture, myocardial infarction, or stroke, or a diagnosis of cancer prior to study baseline, as well as those who reported at study baseline having had a stroke or heart disease. These exclusions were applied because these conditions may influence subsequent physical activity, bone mineral density, body weight, and risk of falls.^25^, ^27^ We further restricted our main analyses to women who reported that they were in good or excellent health, to take account of possible reverse causality; ie, that poor health may cause women to stop or reduce their activities.

### Measures of physical activity

At study baseline (defined as completion of the 3‐year resurvey questionnaire, which was on average 3.3 years after recruitment into the Million Women Study) over 600,000 women responded to the question “About how many hours each week do you spend doing: housework; gardening; walking; cycling; any work or exercise causing sweating or a fast heartbeat”. Except for housework, they were asked to report duration of each activity separately for summer and winter. These questions were structured according to validated, session‐based measures of physical activity questions from both the Active Australia Survey^28^ and the International Physical Activity Questionnaire.^29^ We have previously found a strong association between the number of hours reported on these specific activities and responses given to simple questions on frequency of strenuous and of any activity answered at Million Women Study recruitment.^30^ Women were also asked “Do you belong to or participate in any of the following?”, with activity‐related options including sports club, dancing group, or yoga.

For activities reporting hours each week (housework, gardening, walking, and cycling) we calculated excess metabolic equivalent (MET) hours of activity. This measure represents the energy that would be expended for a given activity above that of the basal metabolic energy expenditure of a person at rest. We have described this method elsewhere.^30^ In brief, multipliers were taken according to the latest information provided by Ainsworth's compendium,^31^ 3.3 for housework, 3.5 for gardening, 3.5 for walking, and 8 for cycling. The value of 1 was subtracted from each multiplier, and then the number of hours spent in each activity was multiplied by this multiplier to provide a value for excess METs for each activity.

### Statistical analyses

All analyses were conducted using Stata, version 14.1 (StataCorporation, Inc., College Station, TX, USA).^32^ We calculated person‐years from the date that women completed the baseline questionnaire. Follow‐up was censored at whichever came first of: date of any fracture (see above in Participants and data section), date of death, date of emigration, or the end of follow‐up, with the last date of follow‐up being March 31, 2015. In cases where more than one fracture was recorded during the same hospital admission, each fracture was included in the analysis of the corresponding specific fracture site. However, multiple upper limb fractures or multiple lower limb fractures occurring during the same hospital admission were counted as only a single event for analyses of the aggregate “upper limb fracture” or “lower limb fracture” outcomes.

Cox regression models were used to calculate relative risks (RRs), and 99% confidence intervals (CIs) for incident fracture at each of the seven sites in relation to various physical activities, with attained age as the underlying time variable. We stratified analyses by study baseline year (≤2001, 2002, 2003, ≥2004) and year of birth (<1935, 1935–1939, 1940–1944, ≥1945), and adjusted for: recruitment region (10 regions), deprivation (quintiles using the Townsend index^33^), educational qualifications (tertiary, secondary/technical, none), height (<155, 155.0–159.9, 160–164.9, 165.0–169.9, or ≥170 cm), smoking status (never, past, current), alcohol consumption (0, 0.1–2.9, 3–6.9, 7–14.9, ≥15 units per week, 1 unit = 10 g), BMI (<20.0, 20.0–22.4, 22.5–24.9, 25.0–27.4, 27.5–29.9, ≥30.0 kg/m^2^), menopausal hormone therapy use (never, past, current), time spent sleeping (≤6.0, 6.1–7.0, 7.1–8.0, >8.0 hours per day), and self‐reported history of: fracture (yes, no), osteoporosis (yes, no), blood clots (yes, no), osteoid/rheumatoid arthritis (yes, no), thyroid disease (yes, no), and diabetes (yes, no). The statistical model for each activity was mutually adjusted for the other activity types defined as follows: walking (≤1.0, >1.0 hours per week), gardening (≤1.0, >1.0 hours per week), cycling (≤1.0, >1.0 hours per week), housework (≤5.0, >5.0 hours per week), participation in yoga (yes, no), participation in dance (yes, no), and participation in sports clubs (yes, no). For the models which used excess MET‐hours, walking, gardening, cycling, and housework were split according to the following excess MET‐hour categories: ≤1.0, >1.0–5.0, >5.0–9.0, >9.0–13.0, >13 excess MET‐hours per week. Using the same categories of excess MET‐hours for each activity type facilitated comparisons of the associations between excess MET‐hours of different activity types and fractures at the various sites.

When more than two groups were compared, RRs were considered as floated absolute risks,^34^ shown with the appropriate group‐specific CI for the log risk. This allowed for valid comparisons to be made between any 2 groups, even when neither group was the reference group. All adjustment variables were reported at study baseline (on average 3.3 years after recruitment into the Million Women Study) except height, year of birth, recruitment region, educational qualifications, and deprivation, which were reported at recruitment. When data were missing for any of the adjustment variables (generally <2% for each variable), women were assigned to an additional “missing” category. We ran sensitivity analyses in which women with missing values were dropped (full case analysis). We ran sensitivity analyses in which there were no restrictions according to health status, and we also ran sensitivity analyses restricting to women who self‐reported being in fair or poor health at baseline, who were excluded from the main analyses. There was no strong evidence for violation of the Proportional Hazards assumption for the various physical activities at each fracture site. The distributions of cycling, walking, gardening, and housework were also presented.

## Results

A total of 371,279 postmenopausal women, aged on average 59.8 ± 4.8 years at baseline and who reported being in excellent or good health, were included in our analyses. The characteristics of these women according to each specific physical activity are shown in Tables 1 and 2. The proportion of current smokers was higher among those who were less likely to participate in the various activities, except for gardening and housework. A higher proportion of women from the most deprived one‐fifth socioeconomically did not participate in yoga or sports club activities, and did fewer hours of gardening per week. Among those with no educational qualifications, a higher proportion did not participate in yoga or sports club activities. Distributions for cycling, walking, gardening and housework are presented in Figs. S1 to S4.

**Table 1:** Characteristics of Women in the Million Women Study According to Walking, Housework, Gardening, and Cycling, Reported at 3‐Year Resurvey

	Walking		Housework		Gardening		Cycling		
Characteristic	Up to 1 hour per week (*n* = 83,505)	More than 1 hour per week (*n* = 287,774)	Up to 5 hours per week (*n* = 84,134)	More than 5 hours per week (*n* = 287,145)	Up to 1 hour per week (*n* = 136,588)	More than 1 hour per week (*n* = 234,691)	Up to 1 hours per week (*n* = 347,155)	More than 1 hour per week (*n* = 24,124)	All women (*n* = 371,279)
Characteristics at baseline									
Age at recruitment (years), mean ± SD	59.5 ± 4.8	59.9 ± 4.8	59.5 ± 4.8	59.9 ± 4.8	59.5 ± 4.8	60.0 ± 4.8	59.9 ± 4.8	59.1 ± 4.5	59.8 ± 4.8
Height (cm), mean ± SD	162.1 ± 6.5	162.5 ± 6.4	162.7 ± 6.5	162.3 ± 6.4	162.1 ± 6.4	162.6 ± 6.4	162.4 ± 6.4	162.8 ± 6.4	162.4 ± 6.4
Weight (kg), mean ± SD	69.0 ± 12.3	67.2 ± 11.0	68.0 ± 11.5	67.5 ± 11.2	68.2 ± 11.8	67.3 ± 11.0	67.7 ± 11.4	66.5 ± 10.4	67.6 ± 11.3
BMI (kg/m^2^), mean ± SD	26.2 ± 4.5	25.5 ± 4.0	25.7 ± 4.2	25.6 ± 4.1	25.9 ± 4.3	25.5 ± 4.0	25.7 ± 4.1	25.1 ± 3.8	25.6 ± 4.1
Alcohol (g/day), mean ± SD	7.0 ± 8.6	7.2 ± 8.4	7.4 ± 8.6	7.0 ± 8.4	7.0 ± 8.6	7.2 ± 8.4	7.1 ± 8.5	7.1 ± 8.2	7.1 ± 8.5
Current smoker (%)	13.0	9.5	9.1	10.7	10.3	10.3	10.5	7.3	10.3
Never smoker (%)	53.0	57.4	55.7	56.6	55.2	57.1	56.3	57.4	56.4
Deprivation: lowest fifth (%)	14.5	13.4	13.8	13.6	17.5	11.4	13.8	11.8	13.7
Never users of HRT (%)	45.2	47.3	44.7	47.4	46.6	46.9	46.7	48.5	46.8
No diabetes (%)	98.2	98.4	98.3	98.4	98.1	98.5	98.3	98.9	98.4
No qualifications (%)	35.2	29.9	25.8	32.6	33.8	29.5	31.1	30.6	31.1
Follow‐up for fracture incidence									
Woman‐years of follow‐up (in millions)	1.0	3.4	1.0	3.4	1.6	2.8	4.1	0.3	4.4
Incident wrist fractures, *n*	1532	5826	1693	5665	2732	4626	6851	507	7358
Incident forearm (not wrist) fractures, *n*	260	978	294	944	517	721	1136	102	1238
Incident humerus fractures, *n*	526	1815	549	1792	963	1378	2190	151	2341
Incident upper limb fracture, *n*	2217	8275	2423	8069	4021	6471	9760	732	10,492
Incident hip fractures, *n*	1094	3260	952	3402	1621	2733	4123	231	4354
Incident femur (not neck) fractures, *n*	174	443	147	470	241	376	580	37	617
Incident lower leg fractures, *n*	288	896	295	889	450	734	1098	86	1184
Incident ankle fractures, *n*	783	2846	903	2726	1373	2256	3403	226	3629
Incident lower limb fracture, *n*	2234	7172	2195	7211	3545	5861	8847	559	9406

BMI = Body mass index; g = grams; HRT = hormone replacement therapy.

Women with missing values were excluded when calculating the means or percentages for that given variable. An average of 12 years follow‐up per woman. Restricted to women reporting that they were in good or excellent health.

**Table 2:** Characteristics of Women in the Million Women Study According to Yoga, Sports Club, and Dance Participation, Reported at 3‐Year Resurvey

	Yoga		Sports club		Dance	
Characteristic	No (*n* = 339,179	Yes (*n* = 32,100)	No (*n* = 286,573)	Yes (*n* = 84,706)	No (*n* = 340,430)	Yes (*n* = 30,849)
Characteristics at baseline						
Age at recruitment (years), mean ± SD	59.9 ± 4.8	59.5 ± 4.7	59.8 ± 4.8	59.8 ± 4.7	59.8 ± 4.8	60.5 ± 4.9
Height (cm), mean ± SD	162.4 ± 6.4	162.8 ± 6.4	162.3 ± 6.5	162.9 ± 6.3	162.5 ± 6.4	161.9 ± 6.4
Weight (kg), mean ± SD	67.9 ± 11.4	64.6 ± 9.5	67.8 ± 11.5	67.0 ± 10.5	67.8 ± 11.4	65.5 ± 10.0
BMI (kg/m^2^), mean ± SD	25.8 ± 4.1	24.4 ± 3.4	25.8 ± 4.2	25.2 ± 3.8	25.7 ± 4.1	25.0 ± 3.6
Alcohol (g/day), mean ± SD	7.0 ± 8.5	8.4 ± 8.3	6.7 ± 8.3	8.6 ± 8.7	7.2 ± 8.5	6.3 ± 7.4
Current smoker (%)	10.8	5.4	11.5	6.2	10.6	6.8
Never smoker (%)	56.2	58.1	56.1	57.4	56.0	61.1
Deprivation: lowest fifth (%)	14.1	8.8	15.0	9.2	13.8	12.6
Never users of HRT (%)	47.3	42.0	47.5	44.3	46.6	48.7
No diabetes (%)	98.3	99.1	98.2	98.8	98.3	98.7
No qualifications (%)	32.5	16.3	34.2	20.6	31.1	31.0
Follow‐up for fracture incidence						
Woman‐years of follow‐up (in millions)	4.0	0.4	3.4	1.0	4.0	0.4
Incident wrist fractures, *n*	6666	692	5649	1709	6713	645
Incident forearm (not wrist) fractures, *n*	1122	116	936	302	1124	114
Incident humerus fractures, *n*	2188	153	1869	472	2155	186
Incident upper limb fracture, *n*	9574	918	8102	2390	9583	909
Incident hip fractures, *n*	4058	296	3571	783	3994	360
Incident femur (not neck) fractures, *n*	591	26	497	120	578	39
Incident lower leg fractures, *n*	1095	89	918	266	1104	80
Incident ankle fractures, *n*	3358	271	2823	806	3348	281
Incident lower limb fracture, *n*	8745	661	7509	1897	8662	744

BMI = Body mass index; g = grams; HRT = hormone replacement therapy.

Women with missing values were excluded when calculating the means or percentages for that given variable. An average of 12 years follow‐up per woman. Restricted to women reporting that they were in good or excellent health.

During an average follow‐up of 12 years per woman (4.4 million person‐years in total), 2341 women were admitted to hospital (either day‐case or overnight stay) for humerus fracture, 1238 for forearm fracture, 7358 for wrist fracture, 4354 for hip fracture, 617 for femur (not neck) fracture, 1184 for lower leg fracture, and 3629 for ankle fracture.

**Figure 1:**
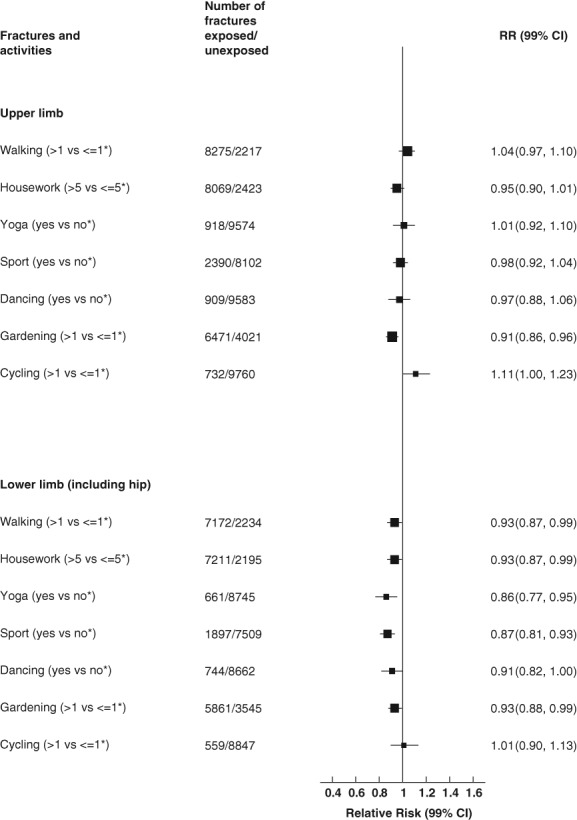
Associations between specific physical activities and upper limb and lower limb (including hip) fractures. *Reference category. Stratified by year of birth and study baseline year, adjusted for attained age, region, deprivation, educational qualifications, height, smoking, alcohol, BMI, menopausal hormone therapy, time spent sleeping, self‐reported history of fracture, osteoporosis, blood clots, osteoid/rheumatoid arthritis, thyroid disease, and diabetes, and mutually adjusted for other activities. Analyses were restricted to women reporting they were in good or excellent health. Women were excluded if they had a hospital record of fracture, stroke, MI, or cancer prior to study baseline, if they self‐reported stroke or heart disease prior to study baseline, or if they were missing information on physical activities of interest or BMI. CI = confidence interval; MI = myocardial infarction; RR = relative risk.

There was heterogeneity across the various activities and risk of fracture of the upper limb (*p* = 0.004). However, statistical heterogeneity across the various activities was not evident at individual sites (*p* = 0.14, humerus; *p* = 0.01, forearm; *p* = 0.31, wrist). Gardening for more than 1 hour per week was associated with reductions in the risk of overall upper limb fracture of 9% (RR = 0.91; 99% CI, 0.86 to 0.96; *p* < 0.0001), of forearm fracture of 24% (RR = 0.76; 99% CI, 0.66 to 0.89; *p* < 0.0001), and of humerus fracture of 17% (RR = 0.83; 99% CI, 0.74 to 0.93; *p* < 0.0001), when compared to gardening less often (Figs. 1 and 3). However, cycling for more than 1 hour per week was associated with an 11% (RR = 1.11; 99% CI, 1.00 to 1.23) increased risk of upper limb fracture when compared to those cycling up to 1 hour per week (*p* = 0.008) (Fig. 1 and Table S3).

**Figure 2:**
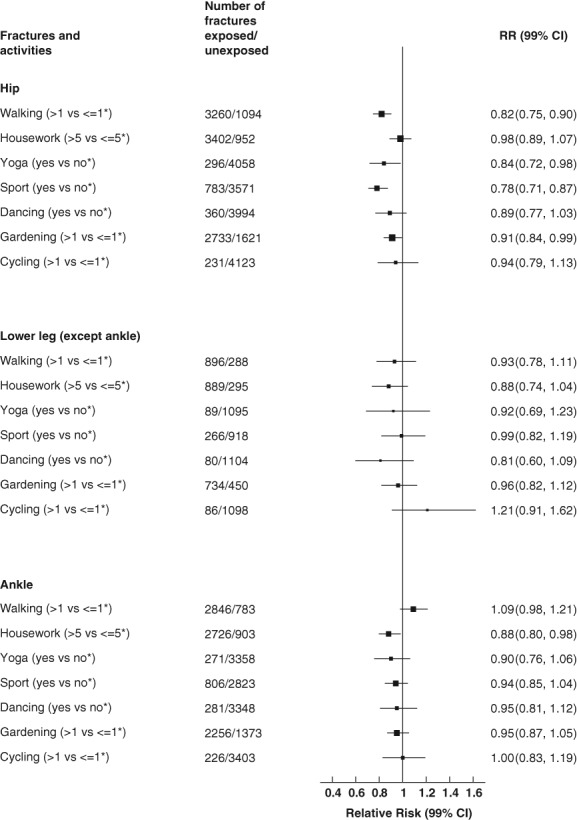
Associations between specific physical activities and lower limb fractures. *Reference category. Stratified by year of birth and study baseline year, adjusted for attained age, region, deprivation, educational qualifications, height, smoking, alcohol, BMI, menopausal hormone therapy, time spent sleeping, self‐reported history of fracture, osteoporosis, blood clots, osteoid/rheumatoid arthritis, thyroid disease, and diabetes, and mutually adjusted for other activities. Analyses were restricted to women reporting they were in good or excellent health. Women were excluded if they had a hospital record of fracture, stroke, MI, or cancer prior to study baseline, if they self‐reported stroke or heart disease prior to study baseline, or if they were missing information on physical activities of interest or BMI. CI = confidence interval; MI = myocardial infarction; RR = relative risk.

There was no heterogeneity in the associations across various activities and fracture risk of the lower limb (including hip) (*p* = 0.31) or of the hip (*p* = 0.02), with physical activity generally associated with a lower fracture risk at these sites. Heterogeneity across various activities was also not evident for femur (not hip) (*p* = 0.37), lower leg (not ankle) (*p* = 0.55), and ankle (*p* = 0.15) fracture. Taking part in yoga was associated with a reduction in risk of lower limb fracture of 14% (RR = 0.86; 99% CI, 0.77 to 0.95; *p* = 0.0001) and of hip fracture by 16% (RR = 0.84; 99% CI, 0.72 to 0.98, *p* = 0.003) (Figs. 1 and 2, Table S3). Participating in yoga was associated with a halving in the risk of femur (not hip) fracture (RR = 0.53; 99% CI, 0.32 to 0.90; *p* = 0.001); however, the CI was wide (Table S4). Participation in sports clubs was associated with a 13% (RR = 0.87; 99% CI, 0.81 to 0.93; *p* < 0.0001) reduction in lower limb fracture and a 22% (RR = 0.78; 99% CI, 0.71 to 0.87; *p* < 0.0001) reduction in hip fracture (Figs. 1 and 2, and Table S3). Walking more than 1 hour per week was associated with an 18% reduction in risk of hip fracture (RR = 0.82; 99% CI, 0.75 to 0.90; *p* < 0.0001) when compared to those walking less often (Fig. 2, Table S3). Gardening more than 1 hour per week was associated with a small reduction in the risk of all lower limb fractures (RR = 0.93; 99% CI, 0.88 to 0.99; *p* = 0.001), and a small reduction in risk of hip fracture (RR = 0.91; 99% CI, 0.84 to 0.99; *p* = 0.003) when compared to those gardening less often (Figs. 1 and 2, and Table S3). Doing housework more than 5 hours per week was associated with a small reduction in the risk of lower limb fracture (RR = 0.93; 99% CI, 0.87 to 0.99; *p* = 0.002,) and a 12% (RR = 0.88; 99% CI, 0.80 to 0.98; *p* = 0.002) reduction in the risk of ankle fracture when compared to those doing less housework (Figs. 1 and 2, Tables S3 and S4). There were no significant associations between any of the activities and the risk of fracture of the lower leg (Fig. 2 and Table S4). Unadjusted risk estimates were similar to adjusted risk estimates.

**Figure 3:**
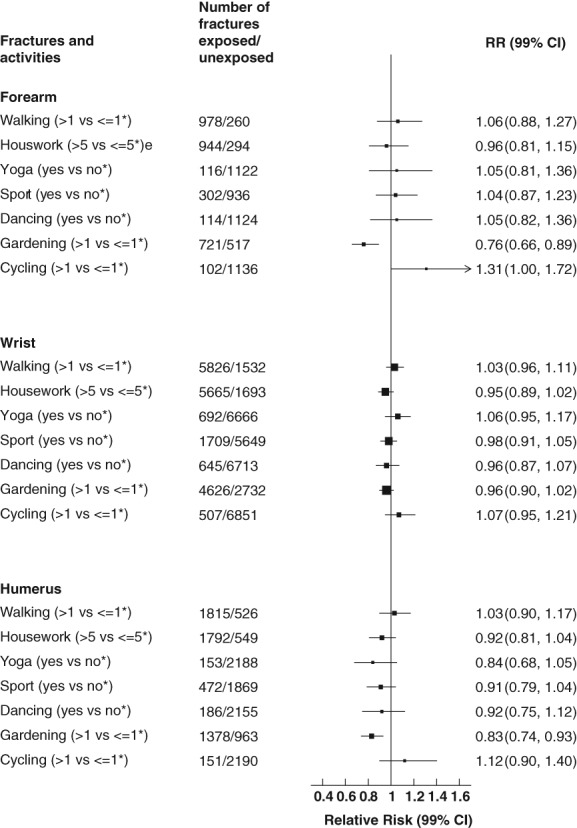
Associations between specific physical activities and upper limb fractures. *Reference category. Stratified by year of birth and study baseline year, adjusted for attained age, region, deprivation, educational qualifications, height, smoking, alcohol, BMI, menopausal hormone therapy, time spent sleeping, self‐reported history of fracture, osteoporosis, blood clots, osteoid/rheumatoid arthritis, thyroid disease, and diabetes, and mutually adjusted for other activities. Analyses were restricted to women reporting they were in good or excellent health. Women were excluded if they had a hospital record of fracture, stroke, MI, or cancer prior to study baseline, if they self‐reported stroke or heart disease prior to study baseline, or if they were missing information on physical activities of interest or BMI. CI = confidence interval; MI = myocardial infarction; RR = relative risk.

When compared to ≤1 excess MET‐hour, gardening for more than 5 excess MET‐hours per week was associated with reductions in risk of upper limb fracture, 15% (RR = 0.85; 99% CI, 0.81 to 0.89) if more than 13 excess MET‐hours per week (Table 3). For individual fracture sites of the arm, gardening was associated with lower risk of both humerus and forearm fracture above 1 excess MET‐hour per week, ranging from 9% to 27% (RR = 0.91; 99% CI, 0.85 to 0.98; to RR = 0.73; 99% CI, 0.66 to 0.81; *p* ≤ 0.001) and 12% to 32% (RR = 0.88; 99% CI, 0.80 to 0.97; to RR = 0.68; 99% CI, 0.57 to 0.82; *p* ≤ 0.001) risk reductions when compared to ≤1 excess MET‐hour for each site respectively (Table 5). More than 13 excess MET‐hours per week of cycling increased the risk of forearm fracture by over 60% (RR = 1.61; 99% CI, 1.27 to 2.04) when compared to ≤1 excess MET‐hour; however, CIs were wide (Table 5).

**Table 3:** Adjusted and Unadjusted Relative Risks, of Upper Limb, Lower Limb (Including Hip), and Hip Fracture in Postmenopausal Women in the Million Women Study According to Excess MET‐Hours of Walking, Housework, Gardening, and Cycling, Reported at 3‐Year Resurvey

	Upper limb			Lower limb (including hip)			Hip		
Parameter	Incident cases	Minimally adjusted	RR fully adjusted RR (99% gsCI)	Incident cases	Minimally adjusted	RR fully adjusted RR (99% gsCI)	Incident cases	Minimally adjusted	RR fully adjusted RR (99% gsCI)
Walking									
≤1 eMET‐hours^a^	1237	1.00	1.00 (0.94–1.06)	1357	1.00	1.00 (0.95–1.06)	689	1.00	1.00 (0.93–1.08)
>1–5 eMET‐hours	2580	1.05	1.03 (0.99–1.08)	2264	0.85	0.88 (0.85–0.92)	1034	0.79	0.82 (0.77–0.87)
>5–9 eMET‐hours	1619	1.06	1.03 (0.99–1.09)	1426	0.85	0.89 (0.84–0.94)	643	0.75	0.78 (0.72–0.84)
>9–13 eMET‐hours	1585	1.04	1.01 (0.97–1.07)	1319	0.79	0.82 (0.78–0.87)	587	0.69	0.71 (0.65–0.77)
>13 eMET‐hours	3471	1.11	1.07 (1.03–1.10)	3040	0.88	0.91 (0.87–0.94)	1401	0.78	0.78 (0.74–0.82)
*p* (heterogeneity)			0.29			<0.001			<0.001
Housework									
≤1 eMET‐hours^a^	812	1.00	1.00 (0.93–1.07)	739	1.00	1.00 (0.93–1.08)	362	1.00	1.00 (0.90–1.11)
>1–5 eMET‐hours	340	0.97	0.93 (0.83–1.03)	362	1.18	1.17 (1.06–1.30)	144	1.08	1.08 (0.91–1.27)
>5–9 eMET‐hours	339	1.02	1.00 (0.90–1.11)	314	1.08	1.08 (0.97–1.21)	130	0.99	1.01 (0.85–1.20)
>9–13 eMET‐hours	932	1.03	1.02 (0.96–1.09)	780	0.98	0.99 (0.92–1.06)	316	0.88	0.90 (0.81–1.00)
>13 eMET‐hours	8069	0.95	0.95 (0.93–0.97)	7211	0.94	0.95 (0.93–0.97)	3402	0.94	0.95 (0.91–0.98)
*p* (heterogeneity)			0.19			<0.001			0.34
Gardening									
≤1 eMET‐hours^a^	2113	1.00	1.00 (0.96–1.05)	2024	1.00	1.00 (0.96–1.05)	952	1.00	1.00 (0.94–1.07)
>1–5 eMET‐hours	3711	0.98	0.96 (0.93–1.00)	3043	0.85	0.88 (0.85–0.91)	1359	0.84	0.87 (0.83–0.92)
>5–9 eMET‐hours	1752	0.95	0.93 (0.89–0.98)	1554	0.87	0.90 (0.86–0.95)	714	0.86	0.88 (0.82–0.95)
>9–13 eMET‐hours	1102	0.92	0.90 (0.85–0.96)	986	0.84	0.87 (0.82–0.93)	469	0.84	0.86 (0.78–0.94)
>13 eMET‐hours	1814	0.88	0.85 (0.81–0.89)	1799	0.87	0.89 (0.85–0.93)	860	0.87	0.86 (0.81–0.92)
*p* (heterogeneity)			<0.001			<0.001			0.009
Cycling									
≤1 eMET‐hours^a^	9220	1.00	1.00 (0.98–1.02)	8417	1.00	1.00 (0.98–1.02)	3947	1.00	1.00 (0.96–1.04)
>1–5 eMET‐hours	196	0.95	0.93 (0.80–1.07)	147	0.82	0.86 (0.73–1.01)	54	0.72	0.76 (0.58–1.00)
>5–9 eMET‐hours	344	1.00	0.98 (0.88–1.08)	283	0.92	0.97 (0.86–1.09)	122	0.92	0.98 (0.82–1.17)
>9–13 eMET‐hours	301	1.05	1.03 (0.92–1.15)	230	0.90	0.94 (0.83–1.07)	93	0.83	0.88 (0.72–1.08)
>13 eMET‐hours	431	1.21	1.17 (1.06–1.29)	329	1.03	1.05 (0.94–1.17)	138	0.97	0.98 (0.83–1.16)
*p* (heterogeneity)			0.02			0.30			0.23

An average of 12 years follow‐up per woman. Restricted to women reporting that they were in good or excellent health. Stratified by year of birth and study baseline year, adjusted for attained age, region, deprivation, educational qualifications, self‐reported health status, height, smoking, alcohol, BMI, menopausal hormone therapy, time spent sleeping, self‐reported history of fracture, osteoporosis, blood clots, osteoid/rheumatoid arthritis, thyroid disease, and diabetes, and mutually adjusted for other activities.

eMET‐hours = excess MET‐hours; gsCI = group‐specific confidence interval; RR = relative risk.

a Reference category.

For lower limb fracture, walking and gardening were both associated with reductions in risk of fracture at energy expenditures >1 excess MET‐hour per week when compared to ≤1 excess MET‐hour per week. For individual fracture sites of the lower limb, walking was associated with reductions in fracture risk at the hip and femur when energy expenditure from the activity was >1 excess MET‐hour per week in comparison to the reference of ≤1 excess MET‐hour per week. These reductions in risk ranged from 18% to 29% (RR = 0.82; 99% CI, 0.77 to 0.87; to RR = 0.71; 99% CI, 0.65 to 0.77; *p* ≤ 0.001) for hip (Table 3) and 35% to 39% (RR = 0.66; 99% CI, 0.54 to 0.81; to RR = 0.61; 99% CI, 0.49 to 0.75; *p* ≤ 0.001) for femur (Table 4). When >1 excess MET‐hour per week was spent gardening, risk of hip fracture was also lowered, in comparison to energy expenditures of ≤1 excess MET‐hour of gardening per week (*p* ≤ 0.001).

**Table 4:** Adjusted and Unadjusted Relative Risks, of Femur, Lower Leg (Excluding Ankle), and Ankle Fracture in Postmenopausal Women in the Million Women Study According to Excess MET‐Hours of Walking, Housework, Gardening, and Cycling, Reported at 3‐Year Resurvey

	Femur			Lower leg (except)			Ankle		
Parameter	Incident cases	Minimally adjusted	RR fully adjusted RR (99% gsCI)	Incident cases	Minimally adjusted	RR fully adjusted RR (99% gsCI)	Incident cases	Minimally adjusted	RR fully adjusted RR (99% gsCI)
Walking									
≤1 eMET‐hours^a^	123	1.00	1.00 (0.83–1.20)	173	1.00	1.00 (0.86–1.17)	438	1.00	1.00 (0.91–1.10)
>1–5 eMET‐hours	135	0.57	0.62 (0.52–0.74)	283	0.82	0.85 (0.76–0.96)	910	1.03	1.05 (0.98–1.12)
>5–9 eMET‐hours	90	0.60	0.66 (0.54–0.81)	194	0.91	0.96 (0.83–1.10)	556	1.03	1.07 (0.99–1.17)
>9–13 eMET‐hours	83	0.55	0.61 (0.49–0.75)	164	0.77	0.82 (0.70–0.95)	536	0.99	1.05 (0.96–1.14)
>13 eMET‐hours	186	0.59	0.63 (0.54–0.73)	370	0.85	0.90 (0.81–1.00)	1189	1.08	1.15 (1.09–1.22)
*p* (heterogeneity)			<0.001			0.29			0.07
Housework									
≤1 eMET‐hours^a^	54	1.00	1.00 (0.76–1.31)	89	1.00	1.00 (0.81–1.23)	265	1.00	1.00 (0.89–1.13)
>1–5 eMET‐hours	20	0.96	0.99 (0.64–1.54)	43	1.06	1.08 (0.80–1.46)	165	1.35	1.31 (1.12–1.53)
>5–9 eMET‐hours	22	1.09	1.15 (0.76–1.74)	47	1.25	1.27 (0.96–1.70)	132	1.16	1.14 (0.96–1.35)
>9–13 eMET‐hours	51	0.92	0.96 (0.73–1.27)	116	1.14	1.16 (0.96–1.39)	341	1.11	1.09 (0.98–1.22)
>13 eMET‐hours	470	0.85	0.86 (0.78–0.95)	889	0.94	0.96 (0.90–1.03)	2726	0.96	0.97 (0.93–1.01)
*p* (heterogeneity)			0.54			0.19			0.001
Gardening									
≤1 eMET‐hours^a^	145	1.00	1.00 (0.84–1.19)	251	1.00	1.00 (0.88–1.14)	765	1.00	1.00 (0.93–1.08)
>1–5 eMET‐hours	196	0.77	0.87 (0.75–1.00)	383	0.83	0.88 (0.79–0.97)	1217	0.86	0.87 (0.83–0.93)
>5–9 eMET‐hours	84	0.66	0.74 (0.60–0.92)	202	0.91	0.96 (0.84–1.10)	628	0.92	0.94 (0.87–1.02)
>9–13 eMET‐hours	52	0.61	0.69 (0.53–0.91)	137	0.96	1.01 (0.85–1.19)	357	0.82	0.84 (0.75–0.93)
>13 eMET‐hours	140	0.94	1.04 (0.88–1.23)	211	0.85	0.90 (0.78–1.03)	662	0.87	0.89 (0.82–0.96)
*p* (heterogeneity)			0.02			0.42			0.02
Cycling									
≤1 eMET‐hours^a^	552	1.00	1.00 (0.91–1.10)	1034	1.00	1.00 (0.93–1.07)	3225	1.00	1.00 (0.96–1.04)
>1–5 eMET‐hours	8	0.71	0.83 (0.41–1.65)	23	0.96	1.02 (0.68–1.54)	68	0.90	0.92 (0.72–1.16)
>5–9 eMET‐hours	20	1.02	1.18 (0.76–1.83)	41	1.03	1.08 (0.80–1.47)	110	0.87	0.89 (0.74–1.07)
>9–13 eMET‐hours	15	0.92	1.04 (0.63–1.73)	33	1.00	1.05 (0.75–1.48)	94	0.90	0.92 (0.76–1.13)
>13 eMET‐hours	22	1.08	1.14 (0.75–1.74)	53	1.31	1.33 (1.02–1.75)	132	1.02	1.04 (0.88–1.23)
*p* (heterogeneity)			0.88			0.41			0.61

An average of 12 years follow‐up per woman. Restricted to women reporting that they were in good or excellent health. Stratified by year of birth and study baseline year, adjusted for attained age, region, deprivation, educational qualifications, self‐reported health status, height, smoking, alcohol, BMI, menopausal hormone therapy, time spent sleeping, self‐reported history of fracture, osteoporosis, blood clots, osteoid/rheumatoid arthritis, thyroid disease, and diabetes, and mutually adjusted for other activities.

eMET‐hours = excess MET‐hours; gsCI = group‐specific confidence interval; RR = relative risk.

a Reference category.

**Table 5:** Adjusted and Unadjusted Relative Risks, of Forearm, Wrist, and Humerus Fracture in Postmenopausal Women in the Million Women Study According to Excess MET‐Hours of Walking, Housework, Gardening, and Cycling, Reported at 3‐Year Resurvey

	Humerus			Forearm			Wrist		
Parameter	Incident cases	Minimally adjusted	RR fully adjusted RR (99% gsCI)	Incident cases	Minimally adjusted	RR fully adjusted RR (99% gsCI)	Incident cases	Minimally adjusted	RR fully adjusted RR (99% gsCI)
Walking									
≤1 eMET‐hours^a^	306	1.00	1.00 (0.89–1.12)	152	1.00	1.00 (0.85–1.18)	841	1.00	1.00 (0.93–1.07)
>1–5 eMET‐hours	577	0.98	1.03 (0.95–1.12)	297	0.97	0.95 (0.84–1.06)	1809	1.08	1.04 (0.99–1.09)
>5–9 eMET‐hours	344	0.92	0.99 (0.89–1.10)	178	0.94	0.92 (0.79–1.06)	1149	1.11	1.05 (0.99–1.11)
>9–13 eMET‐hours	336	0.90	0.97 (0.87–1.08)	198	1.05	1.02 (0.89–1.18)	1129	1.09	1.03 (0.97–1.09)
>13 eMET‐hours	778	1.00	1.07 (1.00–1.15)	413	1.07	1.04 (0.94–1.15)	2430	1.14	1.06 (1.02–1.10)
*p* (heterogeneity)			0.57			0.58			0.66
Housework									
≤1 eMET‐hours^a^	223	1.00	1.00 (0.88–1.14)	91	1.00	1.00 (0.81–1.23)	545	1.00	1.00 (0.92–1.09)
>1–5 eMET‐hours	73	0.80	0.79 (0.63–0.99)	50	1.21	1.10 (0.83–1.45)	235	0.99	0.95 (0.84–1.08)
>5–9 eMET‐hours	75	0.86	0.86 (0.69–1.08)	31	0.80	0.77 (0.54–1.09)	245	1.08	1.06 (0.94–1.21)
>9–13 eMET‐hours	178	0.75	0.75 (0.65–0.87)	122	1.18	1.14 (0.96–1.36)	668	1.09	1.08 (1.00–1.16)
>13 eMET‐hours	1792	0.78	0.79 (0.75–0.83)	944	0.98	1.00 (0.93–1.07)	5665	0.99	0.98 (0.96–1.01)
*p* (heterogeneity)			0.02			0.31			0.18
Gardening									
≤1 eMET‐hours^a^	533	1.00	1.00 (0.92–1.09)	272	1.00	1.00 (0.88–1.13)	1408	1.00	1.00 (0.95–1.06)
>1–5 eMET‐hours	811	0.88	0.91 (0.85–0.98)	447	0.89	0.88 (0.80–0.97)	2605	1.02	1.00 (0.96–1.04)
>5–9 eMET‐hours	373	0.82	0.84 (0.76–0.93)	206	0.84	0.82 (0.72–0.94)	1246	1.01	0.98 (0.93–1.04)
>9–13 eMET‐hours	249	0.84	0.86 (0.76–0.97)	112	0.71	0.68 (0.57–0.82)	780	0.98	0.95 (0.89–1.02)
>13 eMET‐hours	375	0.73	0.73 (0.66–0.81)	201	0.73	0.69 (0.60–0.80)	1319	0.96	0.93 (0.88–0.98)
*p* (heterogeneity)			<0.001			<0.001			0.23
Cycling									
≤1 eMET‐hours^a^	2092	1.00	1.00 (0.95–1.05)	1060	1.00	1.00 (0.93–1.07)	6459	1.00	1.00 (0.97–1.03)
>1–5 eMET‐hours	35	0.80	0.84 (0.61–1.18)	26	1.07	1.03 (0.70–1.51)	145	0.99	0.95 (0.80–1.11)
>5–9 eMET‐hours	63	0.85	0.89 (0.69–1.14)	50	1.22	1.20 (0.91–1.58)	247	1.01	0.97 (0.86–1.10)
>9–13 eMET‐hours	64	1.02	1.09 (0.85–1.39)	33	0.98	0.97 (0.69–1.37)	212	1.05	1.01 (0.88–1.15)
>13 eMET‐hours	87	1.10	1.14 (0.92–1.40)	69	1.65	1.61 (1.27–2.04)	295	1.18	1.12 (1.00–1.26)
*p* (heterogeneity)			0.45			0.009			0.38

An average of 12 years follow‐up per woman. Restricted to women reporting that they were in good or excellent health. Stratified by year of birth and study baseline year, adjusted for attained age, region, deprivation, educational qualifications, self‐reported health status, height, smoking, alcohol, BMI, menopausal hormone therapy, time spent sleeping, self‐reported history of fracture, osteoporosis, blood clots, osteo/rheumatoid arthritis, thyroid disease and diabetes, and mutually adjusted for other activities.

eMET‐hours = excess MET‐hours; gsCI = group specific confidence interval; RR = relative risk.

a Reference category.

Full case analyses of 310,400 women were run; women with missing values for adjustment variables were dropped from the analyses, and results were very similar to the main analyses (Table S1). Sensitivity analyses using 474,388 women (sample not restricted according to self‐reported health) indicated largely similar results to the main analysis (Table S2). Sensitivity analyses restricted to women in poor or fair health generally showed slightly stronger relationships between the various activities and fracture risk than those of the main analysis (Fig. S5), but this could well be because their poor or fair health resulted in women reducing their physical activities.

## Discussion

In this prospective study of 370,000 postmenopausal women who rated their health as good or excellent, physical activities of several different types, including walking, yoga, participation in sports club activities, and gardening, were independently associated with a reduced risk of lower limb and hip fracture. Associations of physical activities with upper limb fracture were more heterogeneous, with gardening being the only activity clearly associated with a significantly reduced risk and cycling associated with a significantly increased risk. For the equivalent excess MET‐hours energy expenditure, gardening showed the greatest reduction in risk for upper limb fracture. For lower limb fractures, walking and gardening were beneficial in terms of fracture risk reductions from excess MET‐hour values >1.

Prior research including both prospective studies and clinical trials has shown the benefits of physical activity in reducing the risk of fractures, especially of more common fractures such as those of the hip.^2^, ^3^, ^4^, ^5^, ^6^, ^7^ Meta‐analyses have shown physical activities can improve balance and reduce falls.^8^, ^9^, ^10^, ^35^ For example, a recent meta‐analysis in community‐dwelling older adults found that the rate of falls was reduced by 21% following exercise programs (pooled rate ratio 0.79; 95% CI, 0.73 to 0.85; *p* < 0.001; *I* ^2^ 47%; 69 comparisons),^9^ and a synthesis of 94 trials concluded weak evidence for moderate improvements in clinical balance outcomes where interventions included either: balance, gait, coordination or functional tasks; strengthening activities, eg, resistance training; or three‐dimensional activities such as dance or yoga.^35^ To our knowledge, ours is the largest prospective study to examine the associations between a range of specific physical activity types and site‐specific fractures in postmenopausal women. Previous prospective studies have shown a similar direction of relationship between walking and fracture risk to our study. The Nurses' Health Study of 61,200 postmenopausal women showed that women reporting walking for 4 or more hours/week had a 41% lower risk of hip fracture (RR = 0.59; 95% CI, 0.37 to 0.94) compared to those who reported walking <1 hour/week.^36^ A smaller prospective study of 9516 postmenopausal woman that compared those who regularly walked for exercise to those who did not, found a nonsignificant relationship with risk of hip fracture (RR = 0.7; 95% CI, 0.5 to 1.0).^6^ A small prospective study (6936 women) compared walking/cycling outdoors for >1 hour/week versus up to 1 hour/week and found a nonsignificant relationship with the risk of wrist fracture, but the CIs were wide.^37^ A prospective study of 9704 postmenopausal women found a 22% reduction (RR = 0.78; 95% CI, 0.62 to 0.99) in the risk of hip fracture when comparing women reporting >9 hours of heavy chores/week to those reporting <5 hours/week.^38^ For most of these studies, numbers were small, with null findings likely to be due to a lack of power.

The potential mechanisms influencing the relationship between physical activity and risk of fracture may oppose each other and are therefore complex, and the evidence on fracture site‐specific mechanisms is sparse. Physical activities aimed at improving muscle strength,^14^ balance,^13^ and coordination may decrease the risk of falls, thereby protecting women from fracture.^9^ Age‐related decreases in bone mineral density may be attenuated through physical activity,^15^, ^16^ but bone strength also depends on various aspects of “bone quality,” including amount of fatigue damage, architecture, and changes in bulk material properties.^39^ Further, while taking part in physical activity, women may be at greater risk of injury; eg, cycling may increase the risk of falling.^17^ Having more cautious physical activity behaviors as a result of a “fear of falling” could also in turn result in an increased risk of falling.^40^ It is possible that the relative strength of these competing risk factures may depend on the type of physical activity and on the fracture site of interest.

The main strengths of this study are its prospective design and large sample size with an average follow‐up of 12 years per woman. Although the information on incident fractures was obtained from objective hospital records, a potential limitation is that less severe fractures did not result in day‐case or overnight admissions and would have been missed. Fractures requiring reduction procedures and/or anesthetics would be included because they require day‐case or overnight stays.^41^ We did not include any fracture codes that specifically indicated trauma in our site‐specific analyses, but it is possible that some fractures related to trauma may have been included. Further, some of the various physical activities were only assessed once at study baseline, meaning that any potential changes in exposure during follow‐up were not captured. The use of self‐reported activity type and duration is a limitation, but objective methods to assess activity type, the aim of this work, were not practical at the point of data collection and are still considerably limited in large‐scale studies. Although the lack of a measure of bone mineral density may be considered a limitation, the use of clinical fracture outcomes, rather than an intermediate measure (such as bone mineral density) is a strength. We accounted for the increased risk of subsequent fracture reported among women with a prior fracture^25^ by censoring at the first occurrence of any fracture. Frail individuals with multiple morbidities may be at an increased risk of fracture^42^ and may participate in less physical activity, but restricting our main analyses to women rating their health as excellent or good should minimize reverse causation bias. Further, restricting to otherwise healthy women is useful in gaining insights into the prevention of ill health later in life. To take account of prior morbidities that might alter physical activity behavior, we excluded women with cancer, myocardial infarction, stroke, or a fracture occurring prior to study baseline.

In conclusion, various physical activities may modify fracture risk in postmenopausal women, but this is dependent on both the fracture site and the type of physical activity. Most activities tended to be associated with a decreased risk of lower limb and hip fracture. For the equivalent energy expenditure, gardening was associated with reductions in risk of both upper and lower limb fracture, and walking with reductions in risk of lower limb fracture. Associations of physical activities with upper limb fracture were more heterogeneous, with gardening associated with a reduced risk and cycling with an increased risk.

## Disclosures

VB is a non‐executive director of the Medicines and Healthcare products Regulatory Agency. MEGA, JL, CJW, BJC, JG, SF and GKR have no conflicts of interest to report.

## Supporting information


**Figure S1:** Distribution of cycling
**Figure S2:** Distribution of walking
**Figure S3:** Distribution of gardening
**Figure S4:** Distribution of housework
**Figure S5:** Associations between specific physical activities and upper limb and lower limb (including hip) fractures for women self‐reporting fair/poor health
**Table S1:** Complete cases analysis for 310,400 women with adjusted and unadjusted relative risks of upper limb, lower limb and hip fracture in postmenopausal women in the Million Women Study according to walking, housework, gardening, cycling, yoga, sports club and dance participation, reported at 3‐year resurvey^**a,b**^
**Table S2:** Adjusted and unadjusted relative risks for 474,388 women, of upper limb, lower limb (including hip) and hip fracture in postmenopausal women in the Million Women Study according to walking, housework, gardening, cycling, yoga, sports club and dance participation, reported at 3‐year resurvey^**a**^
**Table S3:** Adjusted and unadjusted relative risks of upper limb, lower limb (including hip) and hip fracture in postmenopausal women in the Million Women Study according to walking, housework, gardening, cycling, yoga, sports club and dance participation, reported at 3‐year resurvey^**a,b**^
**Table S4:** Adjusted and unadjusted relative risks of femur, lower leg (except ankle) and ankle fracture in postmenopausal women in the Million Women Study according to walking, housework, gardening, cycling, yoga, sports club and dance participation, reported at 3‐year resurvey^**a,b**^
**Table S5:** Adjusted and unadjusted relative risks of forearm, wrist and humerus fracture in postmenopausal women in the Million Women Study according to walking, housework, gardening, cycling, yoga, sports club and dance participation, reported at 3‐year resurvey^**a,b**^
