# Supplementary material for: The Associations Between Seven Different Types of Physical Activity and the Incidence of Fracture at Seven Sites in Healthy Postmenopausal UK Women
Source: J Bone Miner Res. 2019 Nov 15;35(2):277–90. doi: 10.1002/jbmr.3896 (PMC7027536; doi:10.1002/jbmr.3896)
Supplement: Supplementary file 1 — Figure S1: Distribution of cycling Figure S2: Distribution of walking Figure S3: Distribution of gardening Figure S4: Distribution of housework Figure S5: Associations between specific physical activities and upper limb and lower limb (including hip) fractures for women self‐reporting fair/poor health Table S1: Complete cases analysis for 310,400 women with adjusted and unadjusted relative risks of upper limb, lower limb and hip fracture in postmenopausal women in the Million Women Study according to walking, housework, gardening, cycling, yoga, sports club and dance participation, reported at 3‐year resurveya,b Table S2: Adjusted and unadjusted relative risks for 474,388 women, of upper limb, lower limb (including hip) and hip fracture in postmenopausal women in the Million Women Study according to walking, housework, gardening, cycling, yoga, sports club and dance participation, reported at 3‐year resurveya Table S3: Adjusted and unadjusted relative risks of upper limb, lower limb (including hip) and hip fracture in postmenopausal women in the Million Women Study according to walking, housework, gardening, cycling, yoga, sports club and dance participation, reported at 3‐year resurveya,b Table S4: Adjusted and unadjusted relative risks of femur, lower leg (except ankle) and ankle fracture in postmenopausal women in the Million Women Study according to walking, housework, gardening, cycling, yoga, sports club and dance participation, reported at 3‐year resurveya,b Table S5: Adjusted and unadjusted relative risks of forearm, wrist and humerus fracture in postmenopausal women in the Million Women Study according to walking, housework, gardening, cycling, yoga, sports club and dance participation, reported at 3‐year resurveya,b [file JBMR-35-0-s001.docx]

**SUPPLEMENTARY MATERIALS**

**FIGURE S1: Distribution of cycling**

**FIGURE S2: Distribution of walking**

**FIGURE S3: Distribution of gardening**

**FIGURE S4: Distribution of housework**


Table S1: Complete cases analysis for 310,400 women with adjusted and unadjusted relative risks of upper limb, lower limb and hip fracture in postmenopausal women in the Million Women Study according to walking, housework, gardening, cycling, yoga, sports club and dance participation, reported at 3-year resurvey^a,b^

|  | **Upper limb** | | |  | **Lower limb (including hip)** | | |  | **Hip** | | |
| --- | --- | --- | --- | --- | --- | --- | --- | --- | --- | --- | --- |
|  | **Number of fractures exposed/ reference** | **RR minimally adjusted** | **RR**  **fully adjusted RR (99% CI)** |  | **Number of fractures exposed/ reference** | **RR minimally adjusted** | **RR**  **fully adjusted RR (99% CI)** |  | **Number of fractures exposed/ reference** | **RR minimally adjusted** | **RR**  **fully adjusted RR (99% CI)** |
| **Walking (>1 vs <=1^c^)** | 6887/1760 | 1.07 | 1.04 (0.97-1.11) |  | 5881/1720 | 0.92 | 0.94 (0.88-1.01) |  | 2658/817 | 0.85 | 0.85 (0.77-0.95) |
| **P(heterogeneity)** |  |  | 0.17 |  |  |  | 0.04 |  |  |  | <0.001 |
|  |  |  |  |  |  |  |  |  |  |  |  |
| **Housework (>5 vs <=5 ^c^)** | 6695/1952 | 0.93 | 0.95 (0.89-1.01) |  | 5857/1744 | 0.90 | 0.91 (0.84-0.97) |  | 2740/735 | 0.96 | 0.96 (0.86-1.07) |
| **P(heterogeneity)** |  |  | 0.04 |  |  |  | <0.001 |  |  |  | 0.29 |
|  |  |  |  |  |  |  |  |  |  |  |  |
| **Yoga (yes vs no ^c^)** | 786/7861 | 1.05 | 1.01 (0.91-1.11 |  | 568/7033 | 0.86 | 0.88 (0.78-0.98) |  | 249/3226 | 0.84 | 0.85 (0.71-1.00) |
| **P(heterogeneity)** |  |  | 0.89 |  |  |  | 0.003 |  |  |  | 0.01 |
|  |  |  |  |  |  |  |  |  |  |  |  |
| **Sports club (yes vs no ^c^)** | 2010/6637 | 1.00 | 0.97 (0.91-1.04) |  | 1588/6013 | 0.87 | 0.88 (0.81-0.94) |  | 644/2831 | 0.75 | 0.78 (0.70-0.88) |
| **P(heterogeneity)** |  |  | 0.23 |  |  |  | <0.001 |  |  |  | <0.001 |
|  |  |  |  |  |  |  |  |  |  |  |  |
| **Dancing (yes vs no ^c^)** | 752/7895 | 0.99 | 0.97 (0.88-1.07) |  | 601/7000 | 0.87 | 0.91 (0.81-1.01) |  | 288/3187 | 0.87 | 0.89 (0.76-1.05) |
| **P(heterogeneity)** |  |  | 0.45 |  |  |  | 0.02 |  |  |  | 0.07 |
|  |  |  |  |  |  |  |  |  |  |  |  |
| **Gardening (>1 vs <=1^c^)** | 5391/3256 | 0.93 | 0.92 (0.87-0.97) |  | 4772/2829 | 0.92 | 0.93 (0.87-0.99) |  | 2198/1277 | 0.91 | 0.90 (0.82-0.99) |
| **P(heterogeneity)** |  |  | <0.001 |  |  |  | 0.002 |  |  |  | 0.004 |
|  |  |  |  |  |  |  |  |  |  |  |  |
| **Cycling (>1 vs <=1^c^)** | 614/8033 | 1.17 | 1.14 (1.02-1.27) |  | 443/7158 | 0.96 | 1.00 (0.88-1.13) |  | 174/3301 | 0.87 | 0.89 (0.73-1.09) |
| **P(heterogeneity)** |  |  | 0.003 |  |  |  | 0.93 |  |  |  | 0.13 |

RR = Relative risk; CI = Confidence Interval

Stratified by year of birth and study baseline year, adjusted for attained age, region, deprivation, educational attainment, height, smoking, alcohol, BMI, menopausal hormone therapy, time spent sleeping, self-reported history of fracture, osteoporosis, blood clots, osteo/rheumatoid arthritis, thyroid disease and diabetes, and mutually adjusted for other activities

^a^ An average of 12 years follow-up per woman

^b^ A complete case analysis (women with missing values removed) restricted to women reporting that they were in good or excellent health

^c^ Reference category

Table S2: Adjusted and unadjusted relative risks for 474,388 women, of upper limb, lower limb (including hip) and hip fracture in postmenopausal women in the Million Women Study according to walking, housework, gardening, cycling, yoga, sports club and dance participation, reported at 3-year resurvey^a^

|  |  | **Upper limb** | |  | **Lower limb (including hip)** | |  | **Hip** | |
| --- | --- | --- | --- | --- | --- | --- | --- | --- | --- |
|  |  | **RR minimally adjusted** | **RR fully adjusted**  **RR (99% CI)** |  | **RR minimally adjusted** | **RR fully adjusted**  **RR (99% CI)** |  | **RR minimally adjusted** | **RR fully adjusted**  **RR (99% CI)** |
| **Walking (>1 vs <=1^b^)** |  | 1.02 | 1.02 (0.97-1.08) |  | 0.82 | 0.89 (0.84-0.93) |  | 0.74 | 0.80 (0.74-0.86) |
| **P(heterogeneity)** |  |  | 0.33 |  |  | <0.001 |  |  | <0.001 |
|  |  |  |  |  |  |  |  |  |  |
| **Housework (>5 vs <=5 ^b^)** |  | 0.93 | 0.95 (0.90-1.00) |  | 0.90 | 0.92 (0.87-0.97) |  | 0.91 | 0.93 (0.86-1.00) |
| **P(heterogeneity)** |  |  | 0.01 |  |  | <0.001 |  |  | 0.02 |
|  |  |  |  |  |  |  |  |  |  |
| **Yoga (yes vs no ^b^)** |  | 1.03 | 1.00 (0.92-1.09) |  | 0.81 | 0.87 (0.79-0.95) |  | 0.78 | 0.83 (0.72-0.96) |
| **P(heterogeneity)** |  |  | 0.88 |  |  | <0.001 |  |  | <0.001 |
|  |  |  |  |  |  |  |  |  |  |
| **Sports club (yes vs no ^b^)** |  | 0.98 | 0.98 (0.92-1.03) |  | 0.80 | 0.86 (0.81-0.91) |  | 0.70 | 0.79 (0.72-0.87) |
| **P(heterogeneity)** |  |  | 0.28 |  |  | <0.001 |  |  | <0.001 |
|  |  |  |  |  |  |  |  |  |  |
| **Dancing (yes vs no ^b^)** |  | 0.96 | 0.96 (0.88-1.04) |  | 0.84 | 0.92 (0.84-1.00) |  | 0.82 | 0.90 (0.80-1.02) |
| **P(heterogeneity)** |  |  | 0.16 |  |  | 0.01 |  |  | 0.03 |
|  |  |  |  |  |  |  |  |  |  |
| **Gardening (>1 vs <=1^b^)** |  | 0.91 | 0.91 (0.87-0.95) |  | 0.86 | 0.91 (0.87-0.95) |  | 0.83 | 0.89 (0.83-0.95) |
| **P(heterogeneity)** |  |  | <0.001 |  |  | <0.001 |  |  | <0.001 |
|  |  |  |  |  |  |  |  |  |  |
| **Cycling (>1 vs <=1^b^)** |  | 1.13 | 1.12 (1.02-1.22) |  | 0.91 | 0.99 (0.90-1.10) |  | 0.87 | 0.96 (0.82-1.12) |
| **P(heterogeneity)** |  |  | 0.002 |  |  | 0.87 |  |  | 0.48 |

RR = Relative risk; CI = Confidence Interval

Stratified by year of birth and study baseline year, adjusted for attained age, region, deprivation, educational attainment, self-reported health status, height, smoking, alcohol, BMI, menopausal hormone therapy, time spent sleeping, self-reported history of fracture, osteoporosis, blood clots, osteo/rheumatoid arthritis, thyroid disease and diabetes, and mutually adjusted for other activities.

^a^ An average of 12 years follow-up per woman

^b^ Reference category

Table S3: Adjusted and unadjusted relative risks of upper limb, lower limb (including hip) and hip fracture in postmenopausal women in the Million Women Study according to walking, housework, gardening, cycling, yoga, sports club and dance participation, reported at 3-year resurvey^a,b^

|  |  | **Upper limb** | |  | **Lower limb (including hip)** | |  | **Hip** | |
| --- | --- | --- | --- | --- | --- | --- | --- | --- | --- |
|  |  | **RR minimally adjusted** | **RR fully adjusted**  **RR (99% CI)** |  | **RR minimally adjusted** | **RR fully adjusted**  **RR (99% CI)** |  | **RR minimally adjusted** | **RR fully adjusted**  **RR (99% CI)** |
| **Walking (>1 vs <=1^c^)** |  | 1.06 | 1.04 (0.97-1.10) |  | 0.90 | 0.93 (0.87-0.99) |  | 0.82 | 0.82 (0.75-0.90) |
| **P(heterogeneity)** |  |  | 0.13 |  |  | 0.002 |  |  | <0.001 |
|  |  |  |  |  |  |  |  |  |  |
| **Housework (>5 vs <=5 ^c^)** |  | 0.94 | 0.95 (0.90-1.01) |  | 0.92 | 0.93 (0.87-0.99) |  | 0.97 | 0.98 (0.89-1.07) |
| **P(heterogeneity)** |  |  | 0.04 |  |  | 0.002 |  |  | 0.50 |
|  |  |  |  |  |  |  |  |  |  |
| **Yoga (yes vs no ^c^)** |  | 1.05 | 1.01 (0.92-1.10) |  | 0.84 | 0.86 (0.77-0.95) |  | 0.83 | 0.84 (0.72-0.98) |
| **P(heterogeneity)** |  |  | 0.80 |  |  | <0.001 |  |  | 0.004 |
|  |  |  |  |  |  |  |  |  |  |
| **Sports club (yes vs no ^c^)** |  | 1.00 | 0.98 (0.92-1.04) |  | 0.86 | 0.87 (0.81-0.93) |  | 0.75 | 0.78 (0.71-0.87) |
| **P(heterogeneity)** |  |  | 0.29 |  |  | <0.001 |  |  | <0.001 |
|  |  |  |  |  |  |  |  |  |  |
| **Dancing (yes vs no ^c^)** |  | 0.99 | 0.97 (0.88-1.06) |  | 0.87 | 0.91 (0.82-1.00) |  | 0.87 | 0.89 (0.77-1.03) |
| **P(heterogeneity)** |  |  | 0.33 |  |  | 0.01 |  |  | 0.03 |
|  |  |  |  |  |  |  |  |  |  |
| **Gardening (>1 vs <=1^c^)** |  | 0.93 | 0.91 (0.86-0.96) |  | 0.92 | 0.93 (0.88-0.99) |  | 0.91 | 0.91 (0.84-0.99) |
| **P(heterogeneity)** |  |  | <0.001 |  |  | 0.001 |  |  | 0.003 |
|  |  |  |  |  |  |  |  |  |  |
| **Cycling (>1 vs <=1^c^)** |  | 1.14 | 1.11 (1.00-1.23) |  | 0.98 | 1.01 (0.90-1.13) |  | 0.92 | 0.94 (0.79-1.13) |
| **P(heterogeneity)** |  |  | 0.01 |  |  | 0.86 |  |  | 0.40 |

RR = Relative risk; CI = Confidence Interval

^a^ An average of 12 years follow-up per woman

^b^ Restricted to women reporting that they were in good or excellent health

^c^ Reference category

Table S4: Adjusted and unadjusted relative risks of femur, lower leg (except ankle) and ankle fracture in postmenopausal women in the Million Women Study according to walking, housework, gardening, cycling, yoga, sports club and dance participation, reported at 3-year resurvey^a,b^

|  |  | **Femur** | |  | **Lower leg (except ankle)** | |  | **Ankle** | |
| --- | --- | --- | --- | --- | --- | --- | --- | --- | --- |
|  |  | **RR minimally adjusted** | **RR fully adjusted**  **RR (99% CI)** |  | **RR minimally adjusted** | **RR fully adjusted**  **RR (99% CI)** |  | **RR minimally adjusted** | **RR fully adjusted**  **RR (99% CI)** |
| **Walking (>1 vs <=1^c^)** |  | 0.71 | 0.76 (0.60-0.96) |  | 0.89 | 0.93 (0.78-1.11) |  | 1.04 | 1.09 (0.98-1.21) |
| **P(heterogeneity)** |  |  | 0.003 |  |  | 0.28 |  |  | 0.04 |
|  |  |  |  |  |  |  |  |  |  |
| **Housework (>5 vs <=5 ^c^)** |  | 0.87 | 0.87 (0.68-1.11) |  | 0.86 | 0.88 (0.74-1.04) |  | 0.86 | 0.88 (0.80-0.98) |
| **P(heterogeneity)** |  |  | 0.15 |  |  | 0.06 |  |  | 0.002 |
|  |  |  |  |  |  |  |  |  |  |
| **Yoga (yes vs no ^c^)** |  | 0.50 | 0.53 (0.32-0.90) |  | 0.87 | 0.92 (0.69-1.23) |  | 0.87 | 0.90 (0.76-1.06) |
| **P(heterogeneity)** |  |  | <0.001 |  |  | 0.45 |  |  | 0.10 |
|  |  |  |  |  |  |  |  |  |  |
| **Sports club (yes vs no ^c^)** |  | 0.83 | 0.89 (0.68-1.16) |  | 0.98 | 0.99 (0.82-1.19) |  | 0.96 | 0.94 (0.85-1.04) |
| **P(heterogeneity)** |  |  | 0.24 |  |  | 0.88 |  |  | 0.13 |
|  |  |  |  |  |  |  |  |  |  |
| **Dancing (yes vs no ^c^)** |  | 0.67 | 0.70 (0.46-1.08) |  | 0.78 | 0.81 (0.60-1.09) |  | 0.90 | 0.95 (0.81-1.12) |
| **P(heterogeneity)** |  |  | 0.03 |  |  | 0.06 |  |  | 0.41 |
|  |  |  |  |  |  |  |  |  |  |
| **Gardening (>1 vs <=1^c^)** |  | 0.86 | 0.91 (0.73-1.13) |  | 0.93 | 0.96 (0.82-1.12) |  | 0.94 | 0.95 (0.87-1.04) |
| **P(heterogeneity)** |  |  | 0.27 |  |  | 0.50 |  |  | 0.19 |
|  |  |  |  |  |  |  |  |  |  |
| **Cycling (>1 vs <=1^c^)** |  | 1.01 | 1.11 (0.71-1.72) |  | 1.17 | 1.21 (0.91-1.62) |  | 0.97 | 1.00 (0.83-1.19) |
| **P(heterogeneity)** |  |  | 0.56 |  |  | 0.10 |  |  | 0.97 |

RR = Relative risk; CI = Confidence Interval

^a^ An average of 12 years follow-up per woman

^b^ Restricted to women reporting that they were in good or excellent health

^c^ Reference category

Table S5: Adjusted and unadjusted relative risks of forearm, wrist and humerus fracture in postmenopausal women in the Million Women Study according to walking, housework, gardening, cycling, yoga, sports club and dance participation, reported at 3-year resurvey^a,b^

|  |  | **Forearm** | |  | **Wrist** | |  | **Humerus** | |
| --- | --- | --- | --- | --- | --- | --- | --- | --- | --- |
|  |  | **RR minimally adjusted** | **RR fully adjusted**  **RR (99% CI)** |  | **RR minimally adjusted** | **RR fully adjusted**  **RR (99% CI)** |  | **RR minimally adjusted** | **RR fully adjusted**  **RR (99% CI)** |
| **Walking (>1 vs <=1^c^)** |  | 1.07 | 1.06 (0.88-1.27) |  | 1.09 | 1.03 (0.96-1.11) |  | 0.98 | 1.03 (0.90-1.17) |
| **P(heterogeneity)** |  |  | 0.42 |  |  | 0.26 |  |  | 0.56 |
|  |  |  |  |  |  |  |  |  |  |
| **Housework (>5 vs <=5 ^c^)** |  | 0.91 | 0.96 (0.81-1.15) |  | 0.95 | 0.95 (0.89-1.02) |  | 0.91 | 0.92 (0.81-1.04) |
| **P(heterogeneity)** |  |  | 0.59 |  |  | 0.09 |  |  | 0.08 |
|  |  |  |  |  |  |  |  |  |  |
| **Yoga (yes vs no ^c^)** |  | 1.11 | 1.05 (0.81-1.36) |  | 1.13 | 1.06 (0.95-1.17) |  | 0.79 | 0.84 (0.68-1.05) |
| **P(heterogeneity)** |  |  | 0.62 |  |  | 0.17 |  |  | 0.04 |
|  |  |  |  |  |  |  |  |  |  |
| **Sports club (yes vs no ^c^)** |  | 1.08 | 1.04 (0.87-1.23) |  | 1.02 | 0.98 (0.91-1.05) |  | 0.88 | 0.91 (0.79-1.04) |
| **P(heterogeneity)** |  |  | 0.61 |  |  | 0.47 |  |  | 0.07 |
|  |  |  |  |  |  |  |  |  |  |
| **Dancing (yes vs no ^c^)** |  | 1.07 | 1.05 (0.82-1.36) |  | 1.00 | 0.96 (0.87-1.07) |  | 0.88 | 0.92 (0.75-1.12) |
| **P(heterogeneity)** |  |  | 0.60 |  |  | 0.36 |  |  | 0.25 |
|  |  |  |  |  |  |  |  |  |  |
| **Gardening (>1 vs <=1^c^)** |  | 0.78 | 0.76 (0.66-0.89) |  | 0.98 | 0.96 (0.90-1.02) |  | 0.82 | 0.83 (0.74-0.93) |
| **P(heterogeneity)** |  |  | <0.001 |  |  | 0.08 |  |  | <0.001 |
|  |  |  |  |  |  |  |  |  |  |
| **Cycling (>1 vs <=1^c^)** |  | 1.34 | 1.31 (1.00-1.72) |  | 1.12 | 1.07 (0.95-1.21) |  | 1.08 | 1.12 (0.90-1.40) |
| **P(heterogeneity)** |  |  | 0.01 |  |  | 0.14 |  |  | 0.19 |

RR = Relative risk; CI = Confidence Interval

^a^ An average of 12 years follow-up per woman

^b^ Restricted to women reporting that they were in good or excellent health

^c^ Reference category

**Figure S5: Associations between specific physical activities and upper limb and lower limb (including hip) fractures for women self-reporting fair/poor health**

*Reference category; RR = Relative Risk; CI = Confidence Interval

Stratified by year of birth and study baseline year, adjusted for attained age, region, deprivation, educational attainment, height, smoking, alcohol, BMI, menopausal hormone therapy, time spent sleeping, self-reported history of fracture, osteoporosis, blood clots, osteo/rheumatoid arthritis, thyroid disease and diabetes, and mutually adjusted for other activities.

Analyses were restricted to women reporting they were in fair or poor health. Women were excluded if they had a hospital record of fracture, stroke, MI, or cancer prior to study baseline, if they self-reported stroke or heart disease prior to study baseline, or if they were missing information on physical activities of interest or BMI.
